# The Index of Intrusion Control (IIC): Capturing individual variability in intentional intrusion control in the laboratory

**DOI:** 10.3758/s13428-024-02345-z

**Published:** 2024-01-30

**Authors:** Stephanie M. Ashton, Pierre Gagnepain, Per Davidson, Robin Hellerstedt, Akul Satish, Tom Smeets, Conny W. E. M. Quaedflieg

**Affiliations:** 1 Department of Neuropsychology and Psychopharmacology, Maastricht University, Maastricht, the Netherlands; 2 Neuropsychologie et Imagerie de la Mémoire Humaine, Normandie Université, UNICAEN, PSL Research University, EPHE, INSERM, U1077, CHU de Caen, GIP Cyceron, Caen, France; 3 Department of Psychology, Kristianstad University, Kristianstad, Sweden; 4 Centre for Technological Biology, Universidad Politecnica de Madrid, Madrid, Spain; 5 Medical Research Council - Cognitive and Brain Sciences Unit, University of Cambridge, Cambridge, UK; 6 Department of Medical and Clinical Psychology, Center of Research on Psychological disorders and Somatic diseases (CoRPS), Tilburg University, Tilburg, the Netherlands

**Keywords:** Intentional memory control, Intrusions, Index of Intrusion Control (IIC), Think/No-Think task

## Abstract

Intrusive memories can be downregulated using intentional memory control, as measured via the Think/No-Think paradigm. In this task, participants retrieve or suppress memories in response to an associated reminder cue. After each suppression trial, participants rate whether the association intruded into awareness. Previous research has found that repeatedly exerting intentional control over memory intrusions reduces their frequency. This decrease is often summarised with a linear index, which may miss more complex patterns characterising the temporal dynamics of intrusion control. The goal of this paper is to propose a novel metric of intrusion control that captures those dynamic changes over time as a single index. Results from a mega-analysis of published datasets revealed that the change in intrusion frequencies across time is not purely linear, but also includes non-linear dynamics that seem best captured by a log function of the number of suppression attempts. To capture those linear and non-linear dynamics, we propose the Index of Intrusion Control (IIC), which relies on the integral of intrusion changes across suppression attempts. Simulations revealed that the IIC best captured the linear and non-linear dynamics of intrusion suppression when compared with other linear or non-linear indexes of control, such as the regression slope or Spearman correlation, respectively. Our findings demonstrate how the IIC may therefore act as a more reliable metric to capture individual differences in intrusion control, and examine the role of non-linear dynamics characterizing the conscious access to unwanted memories.

**Supplementary Information:**

The online version contains supplementary material available at 10.3758/s13428-024-02345-z.

Intentional memory control allows us to suppress unwanted memories of negative experiences that pose a threat to our well-being (Engen & Anderson, 2018; Gagnepain et al., 2017; Nørby, 2018). This is achieved by executive control mechanisms that can be engaged to stop memory retrieval (Ashton et al., 2020; Levy & Anderson, 2008). Intentional memory control can be investigated via the Think/No-Think paradigm (T/NT; Anderson & Green, 2001). In this task, participants first learn associations between cue–target pairs consisting of words, faces or pictures. After learning, during the T/NT phase, participants either actively retrieve the target in response to the cue (Think trials) or suppress thoughts of the target (No-Think trials). Finally, participants recall all targets (including items that were not presented in the previous phase, which acts as a baseline comparison). In these memory tests, it is typically found that memory performance is lower for memories whose retrieval has been repeatedly suppressed compared to memory for baseline items. The forgetting caused by retrieval suppression is often referred to as suppression-induced forgetting (SIF; for review, see Anderson & Hulbert, 2021).

Suppression-induced forgetting in the T/NT task has been linked to reduced distress from memory intrusions in the following week outside the laboratory (Streb et al., 2016). Outside of the lab, however, people are rarely motivated to retrieve memories that they have actively tried to suppress (Anderson et al., 2016). Therefore, measuring the extent to which memories come to mind despite efforts to suppress them may act as a more applicable measure to real-life memory control, compared to measuring the aftereffects via a recall test. To measure involuntary memory retrieval during intentional suppression in the laboratory, intrusion reports have been incorporated into the T/NT procedure (Levy & Anderson, 2012). On a trial-by-trial basis, participants report to what extent the target came to mind during the presentation of the cue. A failure to prevent retrieval during intentional suppression (i.e., No-Think trial) is classified as an intrusion (Benoit et al., 2015; Hellerstedt et al., 2016; Levy & Anderson, 2012). Generally, the occurrence of intrusions decreases over repeated suppression trials (Benoit et al., 2015; Davidson et al., 2020, Gagnepain et al., 2017; Harrington et al., 2021; Hellerstedt et al., 2016; Levy & Anderson, 2012; Satish et al., 2022; van Schie & Anderson, 2017; although see Nishiyama & Saito, 2022, for null results using a thought substitution strategy). Intrusive memories can be controlled both proactively and reactively (Anderson et al., 2016; Leone et al., 2022), which may underlie distinct forgetting processes. Intrusion reports are therefore key to isolating involuntary memory retrievals and distinguishing between distinct mechanisms of control.

Intrusions measured during intentional memory control have been studied both independently and in relation to later forgetting effects. Although some studies have found a link between intrusions and SIF, where participants who show a greater decline in intrusions also show an increased forgetting effect (Chen et al., 2022; Hellerstedt et al., 2016; Levy & Anderson, 2012; Liu et al., 2020), others have not found such an association (Castiglione et al., 2019; Davidson et al., 2020). Moreover, an overall higher intrusion frequency has been associated with reduced affect suppression (Gagnepain et al., 2017) and SIF (Levy & Anderson, 2012). When measuring the initial intrusion frequency only, no association was found with SIF (Wang & Zhu, 2022; also see Supplementary Table S1 for an overview of results for the association between SIF and intrusion control across datasets included in this paper). These mixed findings may arise from different designs tailored to answer different research questions. For example, different results could arise based on the stimuli used (e.g., words or pictures) and degree of learning based on the associative strength of the cue–target pairs.

The current paper discusses the differences in methodology and analytical approaches used by studies in the past 11 years. The goal of the current study is to examine which linear and non-linear measures best reflect the time course of intrusion control, as observed across multiple studies, in order to identify a universal profile for intentional control of unwanted memories. To compare methods and data between studies, we performed a systematic literature search to obtain an overview of all studies that measure intrusions during retrieval–suppression tasks (see Table 1 for a summary). We then contacted the lead or corresponding authors of all published papers and requested their data. In this paper, we begin by discussing design considerations for several aspects of the T/NT task that are relevant for studies measuring intrusions. Next, by reanalysing datasets from previous studies (see Table 1), we propose a new metric to index intrusion control over time and compare this to previous methods used.

**Table 1:** Overview of methods in intentional memory control studies using intrusion ratings

Studies included in the current paper									
Paper	Conditions	Stimuli		Repetitions	T/NT phase ~ Duration	Breaks between blocks	Intrusion rating	Intrusion analysis	Reference in paper
		Type	Valence						
Ashton et al. (2020)	BS: Stress vs control condition	Word pairs	Negative, autobiographical	12 (2 blocks)	23 min	Duration not specified	3-point scale, 1500 ms	Raw sum score	1
Ashton, Sambeth et al., 2023	BS: Mindfulness training vs control	Object–scene pairs	Neutral	10 (5 blocks)	38 min	45 s break after every block	3-point scale, 3000 ms	IIC	2
Chen et al. (2022)	BS: High vs low cognitive control	Word pairs	Neutral	8 (8 blocks)	38 min	Not specified	3-point scale, 1500 ms	Average frequency of first 4 blocks vs last 4 blocks Intrusion Index: Intrusion frequency of last block – first block / first block	3
Davidson et al. (2020)	BS: Delayed recall with rest vs no rest vs immediate recall	Word–picture pairs	Neutral nouns and neutral or negative images	10 (10 blocks)	37 min	1 m	Y/N response, 1500 ms	Proportionalised slope over 5 blocks	4
Harrington et al. (2021)	BS: Sleep vs sleep deprived	Face–scene pairs	Neutral faces and neutral and negative scenes	10 (5 blocks)	40 min	Not specified	3-point scale, up to 10 s	Proportionalised slope over 5 blocks	5
Legrand et al. (2020)^#^	Two studies: WS: T vs NT vs baseline	Object-scene pairs	Study 1: Neutral vs sadness vs disgust Study 2: Neutral vs disgust	8 (8 blocks)	40 min	Self-paced breaks after every 2 blocks	Study 1: Y/N response, 2000 ms Study 2: 3-point scale, no time limit	RM ANOVA and total frequency	6
Liu et al. (2020)*	WS: T vs NT vs baseline	Map locations – Picture pairs (animal, human, object or scene)	Neutral and negative	10 (5 blocks)	45 min	Not specified	4-point scale, 3000 ms	Slope over 10 blocks using Spearman’s correlation from the average intrusion rating per block	7
Mary et al. (2020)*	BS: PTSD vs trauma exposed vs control	Word–object pairs	Neutral	8 (4 blocks)	32 min	Not specified	Y/N response, 3600 ms	RM ANOVA	8
Nishiyama & Saito (2022)	Two studies : Study 1 : Direct suppression strategy Study 2 : Thought substitution WS : T vs NT vs baseline	Object–scene pairs	Negative, neutral and positive	10 (10 blocks)	30 min	30 s break after every two blocks	3-point scale, 1500 ms	*t*-test comparing intrusion frequency in the first and last block	9
Satish et al. (2022)	WS: T vs NT	Word pairs	Autobiographical: moral vs immoral memories	16 (8 blocks)	40 min	Duration not specified	3-point scale, 1500 ms	Total frequency for part 1 and part 2	10
van Schie & Anderson (2017)	WS: Short vs long NT trial length	Word pairs	Neutral	8 (4 blocks)	39 min	45 s	3-point scale, 1500 ms	RM ANOVA	11
Studies not analysed in the current paper^†^									
Benoit et al. (2015)	WS: T vs NT vs Baseline	Word–picture pairs	Neutral (face vs scene)	10 (5 blocks)	Not specified	Not specified	3-point scale, response time not specified	Proportionalised slope	
Castiglione et al. (2019)	WS: Same probe vs independent probe	Word pairs	Neutral	12 (6 blocks)	38 min	Not specified	3-point scale, 1500 ms	Proportionalised slope	
Castiglione & Aron (2020)	WS: T vs NT vs baseline	Word pairs	Neutral	9 (9 blocks)	38 min	Not specified	3-point scale, 1500 ms	RM ANOVA	
Caudek (2014)	WS: Suppression of self-relevant vs non-self-relevant stimuli	Portrait (photographs of the participant or a stranger)–scene pairs	Negative vs positive targets	Not specified	Not specified	Not specified	Y/N response, duration not specified	Chi-square to compare the probability of suppression compliance between conditions	
Gagnepain et al. (2017)	WS: T vs NT vs baseline	Face–scene pairs	Neutral vs Negative	10 (5 blocks)	40 min	Not specified	3-point scale, up to 10 s	RM ANOVA	
Hellerstedt et al. (2016)	WS: T vs NT vs baseline	Word pairs	Neutral	8 (8 blocks)	60 min	Not specified	3-point scale, 1500 ms	Proportionalised slope	
Levy & Anderson (2012)	WS: Same probe vs independent probe	Word pairs	Neutral	12 (6 blocks)	36 min	Not specified	3-point scale, 1500 ms	Proportionalised slope	
Wang & Zhu (2022)	WS: Thought substitution vs direct suppression vs baseline	Chinese word pairs	Neutral	12 (6 blocks)	25 min	Not specified	Scale and duration not specified	Recorded on the first trial only	

Abbreviations: T = think stimulus type; NT = no-think stimulus type; IIC = Index of Intrusion Control; RM = repeated measures

For papers that included between-subject manipulations, only the control groups were used for comparisons

*These datasets have been used in more recent publications. The dataset used in Mary et al. (2020) was analysed in Leone et al. (2022), the dataset used in Legrand et al. (2020) was also used in Legrand et al. (2022), and data from Liu et al. (2020) were used in Liu et al. (2021)

†These papers were not included in the analysis for one or more of the following reasons: (1) the raw data were not available, (2) the researchers were unable to or did not give permission to share the data, or (3) the design deviations did not allow for comparison with other studies (e.g., Wang & Zhu, 2022, measured intrusions on the first trial only)

## Literature search and inclusion criteria

A systematic literature search was performed through PubMed, Web of Science and PsycINFO (see Supplementary Materials S1 for search terms). We included peer-reviewed empirical studies that were published in English between January 2012 and August 2023. The first author performed the literature search and subsequent review stages. The literature search generated 1328 unique entries (see Supplementary Materials S2 for PRISMA (Preferred Reporting Items for Systematic Reviews and Meta-Analyses) flowchart for an overview of the selection process). The abstracts of all entries were reviewed, and 118 relevant articles were selected. The method sections of these papers were reviewed, and papers were excluded if (a) they did not include the T/NT task (or equivalent, i.e., Imagine/No-Imagine task; Benoit et al., 2016) or (b) did not include intrusion measurements in the retrieval–suppression phase of the T/NT task. This left a final selection of 22 papers (see Table 1).

## Design considerations

### How to measure intrusions: Binary response or a continuum?

At the end of each trial in the retrieval–suppression phase of the T/NT procedure, participants are asked to report whether they thought of the target during the presentation of the cue. One approach is to ask this as a yes/no response. Alternatively, participants may be asked to respond by pressing 1, 2 or 3 on their keyboard (i.e., 1 = never; 2 = briefly; 3 = often). The advantage of the three-point scale is that it increases participants’ mnemonic awareness so as to distinguish between items that they were able to control after an intrusion occurred and those that returned despite repeated efforts to suppress them. By providing a ‘briefly’ option, participants can report short-lived or faint intrusions that they may otherwise not have reported if the response options were more simply yes/no. This approach also enables items with a 2 or a 3 response to be analysed separately (van Schie & Anderson, 2017). In one study, responses were analysed separately using a four-point scale (1 = never; 2 = sometimes; 3 = often; 4 = always; Liu et al., 2020). For imaging studies, isolating different degrees of control could aid in distinguishing between distinct underlying mechanisms of forgetting (e.g., Leone et al., 2022). However, ratings of ‘often’ account for a small proportion of responses (see percentages reported in Harrington et al., 2021; Hellerstedt et al., 2016; Levy & Anderson, 2012); therefore, responses of 2 (briefly) or 3 (often) for No-Think trials are often combined and classified as an intrusion (e.g., Levy & Anderson, 2012). Thus, regardless of the scaling used, the intrusion ratings are generally analysed in a binary fashion.

### Response times

The intrusion rating aims to record a subjective response for whether (or the extent to which) the memory of the target intruded into awareness. Participants are instructed to respond as quickly and accurately as possible, without dwelling on their decision. As the rating is retrospective, the shorter the response window, the closer the timing of the rating is to the occurrence of the intrusion. Response windows range from 1500 ms (Ashton et al., 2020; Davidson et al., 2020; Hellerstedt et al., 2016; Levy & Anderson, 2012; Satish et al., 2022) to 10 s (Gagnepain et al., 2017; Harrington et al., 2021). Although the rating pertains to the trial that has just passed, longer intrusion response windows may increase the likelihood of participants unintentionally recalling the associated memory.

Large variability in the length of response time windows between studies may yield different reaction times between participants in different studies or within the same study, which may influence the outcome or interpretation of results. Ratings closer in time to when an intrusion occurred may not be comparable to ratings given later in the response window, as perhaps the clarity or awareness of the intrusion may differ. Additionally, if participants progress to the next trial as soon as a response is recorded, response times may differ within studies. Participants could then vary in the time it takes to complete the task, which could result in short intervals between suppression trials, or fatigue if participants make use of the full response window. Fatigue (Harrington et al., 2021) and duration of suppression periods (i.e., the length of time the reminder cue is presented; van Schie & Anderson, 2017) have been found to reduce memory control abilities. These factors should be taken into consideration when determining the duration of the T/NT phase, based on the number of stimuli cued and trial length. We recommend an intrusion response window of 3000 ms to reduce variability in trial length and to keep the interval relatively short. If the participant responds before the end of the response window, then a black screen can be presented for the remainder of the time until the next trial begins (see, e.g., Ashton, Sambeth et al., 2023). Presenting a black screen, rather than keeping the intrusion rating on the screen, prevents the participant from continuing to monitor the memory after they have assessed it. However, missing trials may occur in a short response window and should be considered during analysis (see Section “Calculating intrusion frequencies”).

## Considerations for analysis

### Calculating intrusion frequencies

When measuring intrusions with the T/NT task, typically only items that have been learned in phase 1 of the task are used in subsequent analyses^1^. Several outcome measures can be used to analyse intrusions. One is the total sum of intrusions overall, which has been reported using raw scores (Ashton et al., 2020) or expressed as percentages (Gagnepain et al., 2017; Levy & Anderson, 2012; Satish et al., 2022). Another measure is to calculate intrusions for each block of the T/NT task. Both measures can be expressed as absolute or proportionalised scores.

When calculating either the total sum or intrusions per block, the number of valid trials should also be taken into account, i.e., removing trials without a response. Without a rating, it is not possible to determine the success of the trial. If missing trials are coded as 0 (and are therefore considered a non-intrusion), then the results may not accurately reflect intrusion control. Out of the published studies in Table 1, only two reported how missing data were accounted for in the analysis (Nishiyama & Saito, 2022; van Schie & Anderson, 2017).

Our reanalysis of the data made available per trial [1, 2, 5, 7, 9, 10, see Table 1], revealed that the number of missing intrusion ratings was low (1500 ms response window [1, 9, 10]: *M* = 2.91%; 3 s response window [2]: *M* = 0.39%; [7]: *M* = 0%; 10 s response window [5] : *M =* 0.02%). We recommend that not only should missing trials be considered when calculating the total intrusion frequency (e.g., Nishiyama & Saito, 2022), but also the distribution of these missing trials across the task. To avoid missing trials, especially in the first block of the task, practice trials are key for ensuring participants know how to respond accurately for the intrusion rating. We suggest a minimum cut-off of 75% of valid trials per block, as this ensures that there are enough trials to reliably calculate the percentage of intrusions per block. This minimum cut-off is based on the study that had the fewest number of items in Table 1 (5 items, Satish et al., 2022), which would be a strict criterion that allowed for only one missing trial for each condition per block. Depending on the number of items, a higher cut-off than 75% could be applied, as a quarter of missing trials per block could lead you to question your participant’s compliance with task instructions. The cut-off should be applied separately per condition (i.e., Think and No-Think trials) and, if present, per stimulus type (e.g., neutral versus emotional). The data of participants who do not meet this cut-off should be excluded from analysis.

## Measuring intrusion control across suppression attempts through a single value

**Fig. 1:**
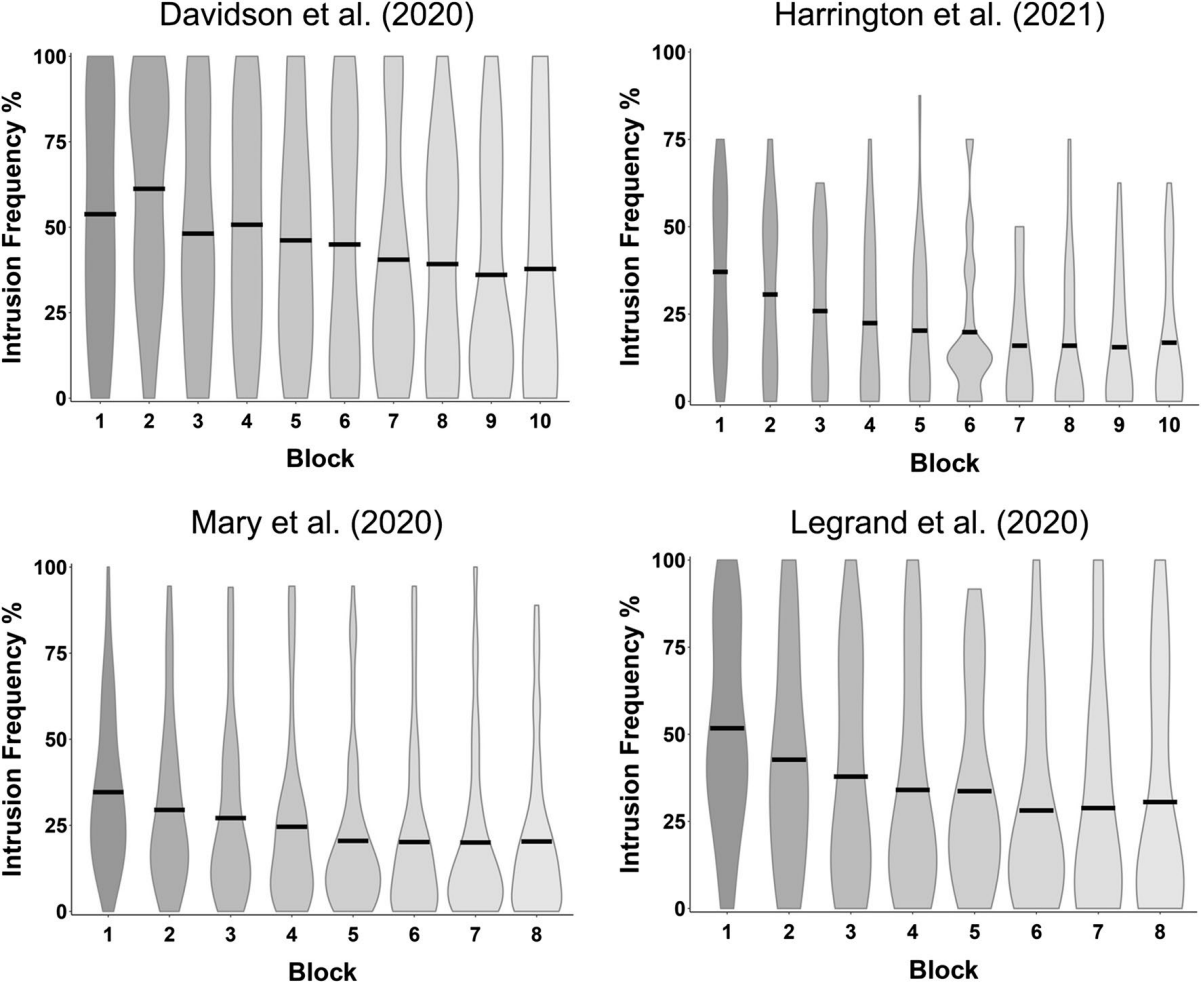
Variability in intrusion control across repeated suppression between studies. The violin plots display data from four studies (Davidson et al., 2020: *n* = 17; Harrington et al., 2021: *n* = 29; Mary et al., 2020; *n* = 73; Legrand et al., 2020 [study 2]: *n* = 24). The means (indicated by the black bar) and the distribution of the data demonstrate large variability within each block of the T/NT phase both within and between studies. Each graph was made for intrusion responses to neutral stimuli

The variation in intrusion proportions across repeated suppression provides important information to characterise the efficiency of the underlying control process and its improvement with practice. Repeated-measures analysis of variance (ANOVA) on the frequency of intrusions reported across the blocks of the T/NT phase consistently report that the frequency of intrusions decreases over time (for review, see Hu et al., 2017). However, as shown in Fig. 1, a large variability in intrusion frequencies can be observed per block across studies. Summarizing those changes using a single index of intrusion control can facilitate the comparison between measures recorded after suppression, or with other behavioural, neural or psychopathological markers collected at the individual level. Producing a single index for intrusion control could also allow for group comparisons by using a median split approach (Benoit et al., 2015).

### The linear slope approach

Previous research has indexed intrusion control using linear approaches to quantify time-dependent changes. In this approach, a regression model with T/NT blocks as the predictor and block by block intrusion proportions as the dependent variable is fitted for each participant, producing an individualised beta slope value. The beta slope quantifies the overall change between the first and last block (Hellerstedt et al., 2016; Levy & Anderson, 2012). The strength of the linear component of the trend acts as the reflective measure of successful intrusion control. As such, the slope approach prioritises the overall downward decline between the initial and final intrusion frequency, rather than the aggregate success across trials.

To account for the fact that the possible change in intrusions is dependent on the initial frequency of intrusions, previous studies have proportionalised the slope measure by either dividing the value by the frequency in the first trial or block (Benoit et al., 2015; Castiglione et al., 2019; Harrington et al., 2021; Levy & Anderson, 2012) or by setting the frequency of the first trial or block to 100% (Davidson et al., 2020)^2^. By proportionalising the value, the steepness of the slope is not determined by the initial frequency, and the participant’s baseline level of exerting intentional control is corrected for. The disadvantage of this approach is that proportionalisation tends to produce extreme values and induces skewness in data distribution. The main disadvantage of the linear slope is that it does not capture non-linear changes across repeated suppression attempts. Yet, those non-linear changes may reflect important dynamic shifts that occurs at the attentional or control level (Song et al., 2021). Here, we performed a mega-analysis^3^ across studies to assess the importance and universality of this non-linear term. We designed three linear mixed-effect models and compared models including or not including this non-linear term. The dependent variable is the total intrusion frequencies per block of the T/NT task. To account for the variability of intercept and slope across studies and participants, the first model included the suppression repetition as the fixed effect, and study and participant as random effects. The second model was identical, but included an additional quadratic term (the square of T/NT blocks) to capture non-linear dynamics. The third model was identical to the second, but the non-linear term was modelled using the log of T/NT blocks instead of a quadratic term. After fitting each model separately, we performed a likelihood-ratio (LR) test to compare the goodness of fit of the linear model against the two competing non-linear statistical models.

The results were unambiguous and favored models including non-linear terms (quadratic model: LR = 237, Δ-_degree of freedom_ = 7, *p* < .00001; log model: LR = 359, Δ-_degree of freedom_ = 7, *p* < .00001). Interestingly, the modelling of the non-linear term using the log function was preferred over its quadratic expression (LR = 122.7, Δ-_degree of freedom_ = 0, *p* < .00001). Several studies have condensed the blocks of the T/NT task (e.g., 8 or 10 repetitions) by averaging them into fewer blocks (e.g., between 4 or 5), thus increasing linearity but losing significant data points and thus information on individual variability. Despite this, when the data in our model comparison were condensed into fewer blocks, the results remained unchanged, and the same pattern emerged in favour of the model including both a linear and a log term.

### Index of intrusion control (IIC)

**Fig. 2:**
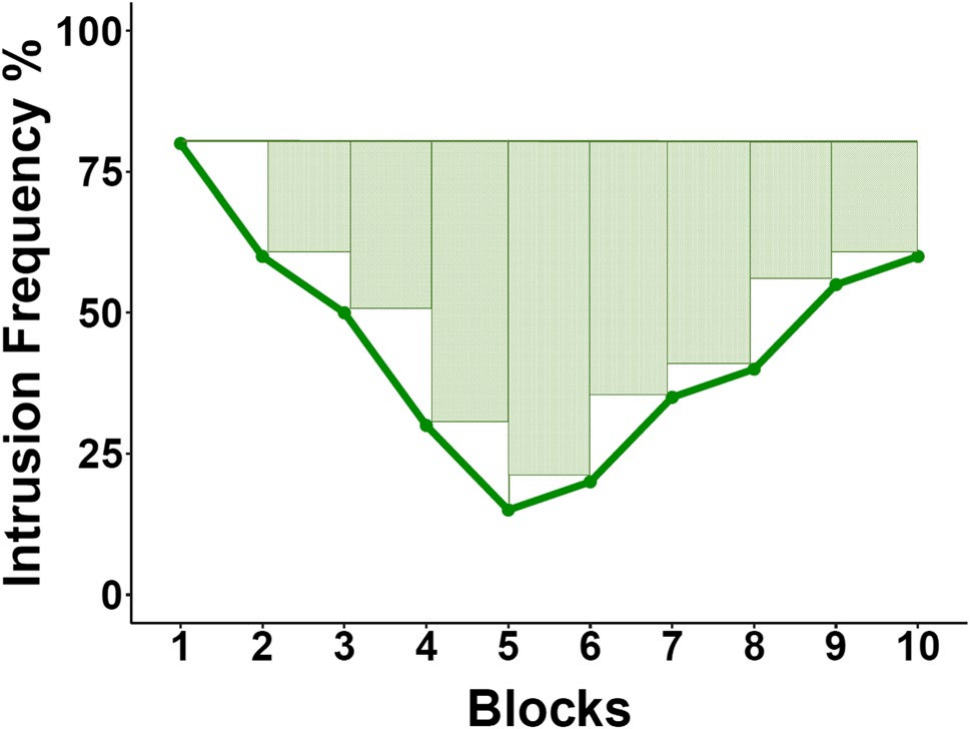
Annotated line graph of example intrusion frequencies over 10 blocks to demonstrate the formula used to calculate the Index of Intrusion Control. The IIC calculates the total area within the repeated measures by combining the area between each time point (crossed rectangles) and the increase or decrease between time points (white triangles)

The findings from the mega-analysis suggest that both linear and non-linear components are important to capture the change in intrusion proportions across T/NT blocks. To this end, we propose an alternative measure to determine the change in the frequency of intrusions across blocks of the T/NT phase that can capture both linear and non-linear dynamics into a single index. The Index of Intrusion Control (IIC) uses the formula to calculate the area under the curve (AUC; Pruessner et al., 2003). The AUC can be applied to repeated measurement data and can be used to capture either the distance from the ground (AUCg; i.e., the total response) or the change over time in reference to the baseline measurement (AUCi, with respect to increase). These formulas are commonly used in endocrinological studies to estimate secretion of hormones over a time period (Fekedulegn et al., 2007; Zorn et al., 2017) or in pharmacological studies to evaluate plasma concentrations of a drug across time after dosage (Scheff et al., 2011). The IIC (visualised in Fig. 2) uses the AUCi, which considers the initial intrusion value and dynamically captures variability with respect to adjacent data points. The IIC for a T/NT phase with 10 blocks is calculated as follows:

$$\frac{(Block1+Block10)}{2}+\left(\sum_{n=2}^{9}Blockn\right)-\left(9\times Block1\right)$$

A syntax for this formula with a breakdown of each step of the calculation is available at the Open Science Framework (https://osf.io/mr7xb/?view_only=14e5b5d60ba847248132b49e34ff061b.). Please see Pruessner et al. (2003) for a comprehensive description for each term of the formula. Increasingly negative values indicate a larger decrease in intrusions over time (i.e., IIC decrease), whereas positive values indicate either no change (i.e., a value of 0) or an increase in intrusions over time (i.e., IIC increase). In theory, the AUCg could be used to look at the total intrusion frequency over time. However, calculating the total frequency as a single percentage is easier to interpret (Gagnepain et al., 2017; Levy & Anderson, 2012; Satish et al., 2022).

**Fig. 3:**
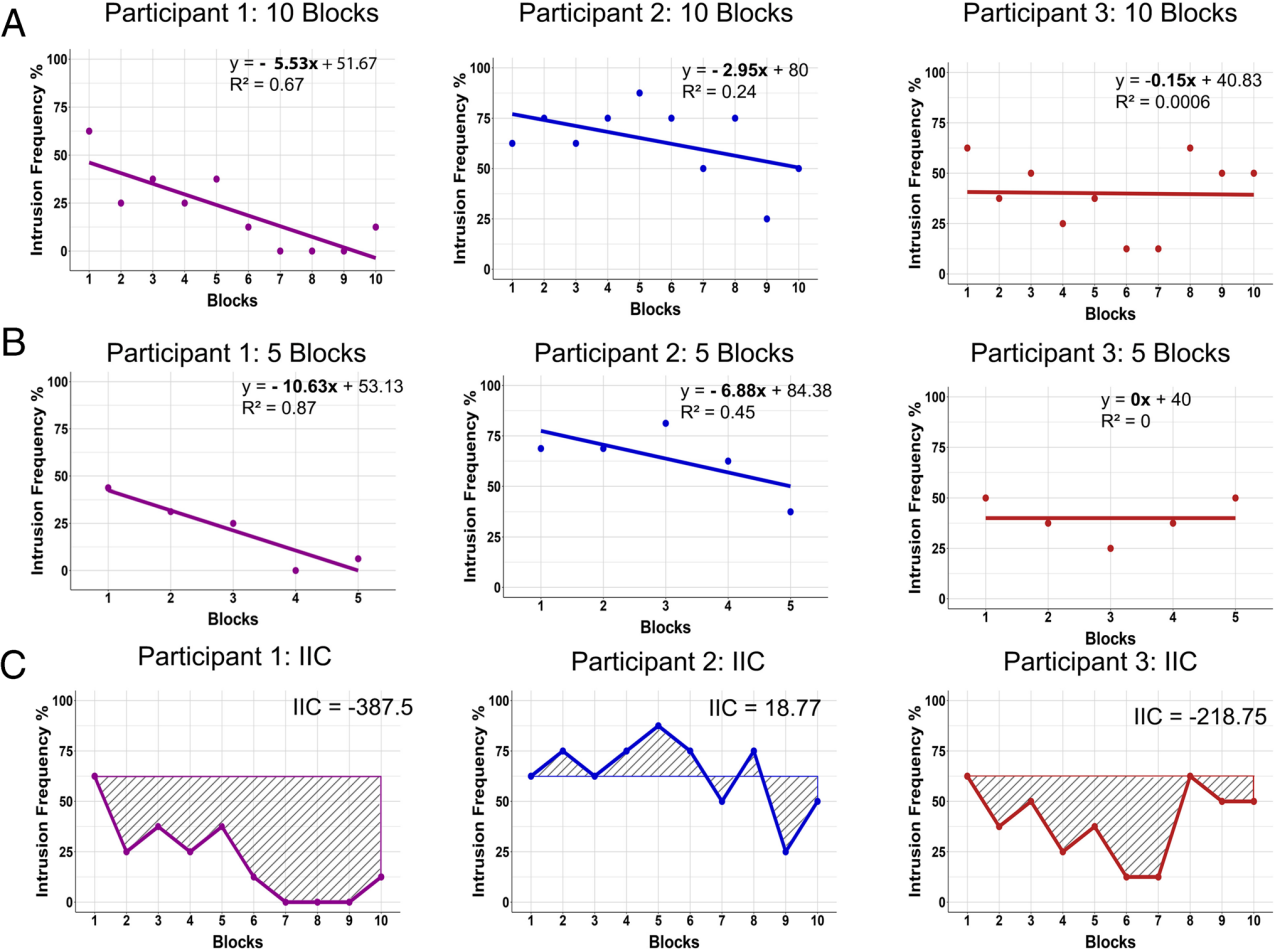
Example data presenting intrusion frequencies across 10 vs 5 blocks vs IIC for three participants from Harrington et al (2021). **A** The intrusion frequencies across 10 blocks. For participant 1, the slope has an increased fit (*R*^2^ = .67) compared to participants 2 and 3, who have a low fit or no fit for the observed data (*R*^2^ =.24 and *R*^2^ =.00, respectively). **B** The same data condensed into five fewer blocks. For participants 1 and 2, condensing the data increases linearity and improves the fit of the slope when compared to 10 blocks. For participant 3, the slope fit does not improve. **C** The IIC. The area used to calculate the value is blocked out in grey

Figure 3 presents a comparison of the slope versus the IIC for three participants of Harrington et al. (2021). The upper panel presents the linear slope value for each participant across 10 trials. In some cases, the slope function may be fitting (e.g., participant 1), while in other cases, it less accurately models the data (e.g., participants 2 and 3). The middle panel of Fig. 3 shows that the slope fit also depends on how the data are processed (i.e., individual repetitions or in blocks). Creating condensed blocks might make the data more linear in general, but does not result in a better fit for all participants (e.g., participant 3). The lower panel in Fig. 3 visualises the IIC. Note that the slope measure would judge participant 2 to have enhanced intrusion control compared to participant 3, whereas the IIC would conclude the opposite. In the case of participant 2, the slope demonstrates a decline in intrusion frequency over time. Looking at the raw data points (in which the repeated measures are connected with the IIC), this participant shows an increase in intrusion frequency until block 7, with a steep decline again at block 9, before returning close to their baseline frequency. In contrast, the intrusion frequency of participant 3 declines from the initial intrusion frequency until block 7, before making a return to their baseline frequency. In both cases, the participant ends on a similar intrusion frequency to when they began the task, but the IIC captures additional and sustained changes over time that are missed by the slope.

Capturing how suppression changes over time provides more insight into individual differences in the ability to control intrusions, which the slope and IIC achieve in different ways. Compared to the slope measure, the IIC takes all data points into account and the difference between each subsequent time point. This might produce additional insightful variability which could better capture individual differences in intrusion control than linear methods or methods comparing the first and last half of the task (e.g., Chen et al., 2022) or the first and last block (e.g., Nishiyama & Saito, 2022).

### Simulation of intrusion control

**Fig. 4:**
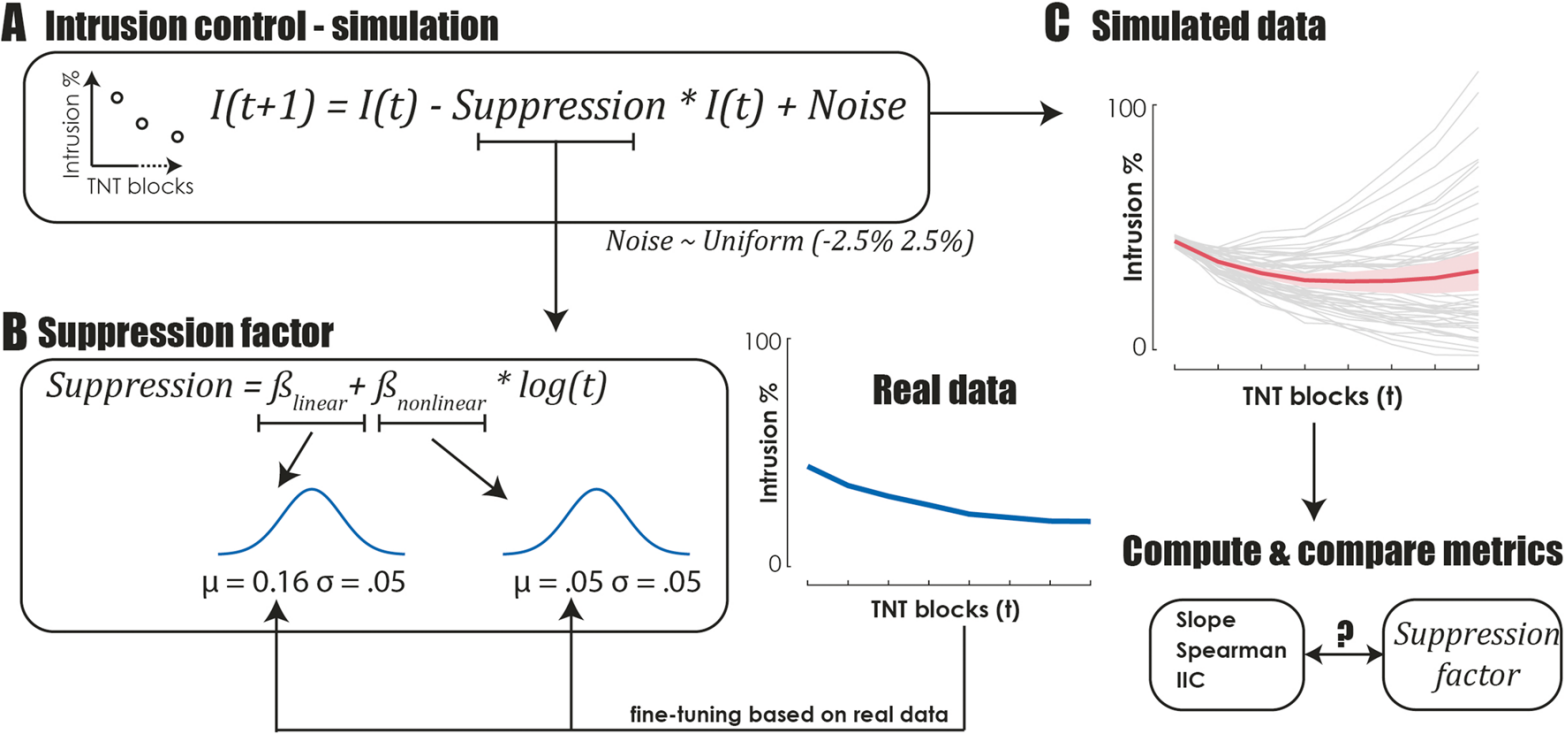
Outline of the model used to simulate intrusion control. The formula (**A**) simulates the frequency of intrusions (I) across T/NT blocks (t) using both linear (β_linear_) and log-non-linear (β_nonlinear_) terms. The sampling distribution of these suppression terms is calibrated from the combined real datasets (**B**). Data are then simulated for 50 participants across 8 blocks (**C**) after adding 5% uniform noise. The various metrics of intrusion control are computed and compared to the suppression factor used to generate the data to understand which metric best captures intentional suppression of intrusions

To further demonstrate and validate the additional values provided by the IIC, we simulated intrusion data using a suppression model that generated intrusion frequencies across T/NT blocks using a combination of linear and log-non-linear terms (see Fig. 4). The linear term describes the amount of control applied during each block, reflecting the stable decrease in intrusions that occurs after each suppression attempt. The non-linear term describes the reduction in intrusion control, which gradually increases across the blocks according to a log function. We first fit this generative model to the average intrusion frequency across all studies and participants. This allowed us to simulate data around this universal and generic intrusion pattern observed across all studies. To do this, we sampled the two suppression parameters from a normal distribution centred around these generic parameters, generated intrusion frequencies using these sampled parameters, and added 5% noise. We performed this simulation for 50 virtual participants and, for each, computed the slope, the IIC and (as an alternative method, see Liu et al., 2020) the Spearman correlation that are associated with these synthetic intrusion data. The Spearman correlation was added to gain insight into how the IIC behaves against another non-linear marker of intrusion control. For each index, we then computed the percentage of variance explained (*R*^2^) by the true generative suppression parameters. We repeated this virtual experiment 1000 times to obtain bootstrapped confidence intervals of *R*^2^ estimates.

Results demonstrated that the slope explained 88% of the variance (95% CI = [78–93%]), while the IIC and Spearman explained 94% (95% CI = [90–96%]) and 56% (95% CI = [37–71%]), respectively. Importantly, the IIC significantly outperformed both the slope (95% CI of the difference = [2–11%]) and the Spearman correlation (95% CI of the difference = [23–57%]) as indexes of control. These findings indicate that the IIC is better at capturing both the linear and non-linear dynamics associated with intrusion control when compared to the standard slope index or Spearman correlation as another non-linear index.

## Discussion

Our findings from the mega-analysis revealed that models including both a linear and non-linear log term best captured the change in intrusions over repeated suppression attempts. In order to capture both linear and non-linear dynamics in a single index, we put forward the IIC. This metric takes intrusion frequencies from all blocks of the T/NT into account and the difference between each subsequent time point. Our simulation analysis revealed that the IIC best captured both linear and non-linear dynamics of intrusion control when compared to other indexes, i.e., the regression slope or Spearman’s correlation. The IIC has the potential to produce additional insightful variability which may better capture individual differences in intrusion control compared to approaches used previously.

Several aspects of brain cognition that interact with inhibitory control mechanisms might introduce non-linearity during intrusion control. For instance, intrusions might depend on the contribution of two separable memory retrieval processes, familiarity and recollection, reflecting the (linear) strength of the memory traces and a threshold-based retrieval process, respectively (Yonelinas et al., 2010). From this perspective, non-linear patterns during intrusion control can emerge, whereby inhibition targets are selectively increased and gradually modulate hippocampus-dependent threshold processes. Furthermore, it has been argued that conscious access to our memories is achieved through activation of a non-linear network, combining other cognitive processes such as attention and evaluation (see global workspace theory reviewed in Seth & Bayne, 2022). Moreover, considering the non-linear components of intrusion control could allow us to capture other time-changing effects, such as criterion shift (Layher et al., 2020), attentional drift (Sali et al., 2016), motivation or cognitive fatigue (Westbrook & Braver, 2015). These factors may modulate the proportion of intrusions and, given that the combination of these effects is probably non-linear, the IIC could also capture those effects. In other research designs, such as ecological momentary assessment (Kleim et al., 2013), the IIC could also arguably act as a more accurate model for capturing the experience of intrusions in real life and their temporal dynamics within or across days.

In addition to using the IIC as a single index, regression modelling could be used to study linear and non-linear components of intrusion control separately. This could be used to determine which components better capture SIF, thereby providing more insight into individual differences in the ability to control intrusions and subsequently forget. On the one hand, the linear approach would judge successful intrusion control as a greater decline in intrusions between the beginning and end of the retrieval–suppression phase. These accumulating inhibitory aftereffects may accurately reflect the ability to suppress, which is the same logic that is argued to result in SIF. On the other hand, the IIC takes into account the aggregate success across all trials and their total retrieval-suppression performance, which may act as a better indicator of SIF.

In addition to the choice of analysis, design choices for the T/NT can influence the interpretation of intrusion control. Given that the ratings are subjective, the understanding and interpretation of instructions for how to suppress and report intrusions could greatly influence results. Although beyond the scope of this paper, studies should make their instructions publicly available, including what is read by the participant and what is verbally discussed during practice and the T/NT task itself (e.g., Ashton, Sambeth et al., 2023, Ashton, Smeets et al., 2023; Liu et al., 2020). Other design aspects, such as the choice of stimuli, may also influence the ability to suppress. During learning, some pairs may be easier to associate together, which could make them more difficult to later suppress than pairs that had weaker associations. To account for this, researchers may counterbalance the pairs across conditions (i.e., Think, No-Think and baseline). When creating a single index, researchers may then *z*-normalise the indexes per counterbalancing condition to account for item effects (Nardo & Anderson, 2023). However, counterbalancing items across conditions may not always be possible, especially with complicated designs that have several counterbalancing conditions. Alternatively, if the design allows, researchers may randomise allocation of pairs to Think/No-Think/baseline conditions, which would avoid the need to standardise scores at the cost of increasing noise from item effects. For an extensive review of other methodological considerations and standardised practices for running a T/NT task, please see Nardo and Anderson (2023).

Moreover, it should be noted that studies that have been designed with the intention to investigate intrusion control as their primary outcome may not be optimised to also investigate intentional forgetting (i.e., SIF). Cue–target pairs in some studies were purposefully over-trained in order to increase the likelihood of intrusions (Gagnepain et al., 2017; Harrington et al., 2021; Legrand et al, 2020). On the one hand, forming strong associations between cue–target pairs (i.e., via high learning criteria or overtraining) more closely resembles the formation of real and salient memories, as these are rehearsed in real life. On the other hand, overtraining can result in expected ceiling effects on the final recognition or recall test (e.g., Gagnepain et al., 2017; Harrington et al., 2021). We recommend that studies preregister whether their primary research question is to investigate intrusion control. If this is the case, the design should be optimised to strengthen memory associations, and thus inclusion of the final recall task and analysis of SIF may not be an appropriate or insightful addition (see Castiglione & Aron, 2020).

In sum, the IIC provides an alternative metric for researchers to consider that can capture the non-linear components of intrusion control, as observed across multiple studies. This may act as a more reliable metric to capture subtle individual differences in the ability to exert control over unwanted memories.

### Supplementary Information

Below is the link to the electronic supplementary material.

Supplementary file1 (DOCX 57 KB)

## Data Availability

Available via https://osf.io/mr7xb/?view_only=14e5b5d60ba847248132b49e34ff061b.

## Footnotes

1 As with SIF data, researchers could opt to analyse both the conditionalised and unconditionalised intrusion data (i.e., items that had not been learned in phase 1); see, e.g., Castiglione et al. (2019).

2 In one exception, the slope was proportionalised by dividing each block by the frequency of the first block and then calculating the slope (Hellerstedt et al., 2016).

3 From the available datasets in Table 1, only the dataset of Liu et al. (2020) was not included, due to the different scaling of the intrusion rating.
