# Supplementary material for: The Index of Intrusion Control (IIC): Capturing individual variability in intentional intrusion control in the laboratory
Source: Behav Res Methods. 2024 Jan 30;56(4):4061–72. doi: 10.3758/s13428-024-02345-z (PMC11133188; doi:10.3758/s13428-024-02345-z)
Supplement: Supplementary file 1 — Supplementary file1 (DOCX 57 KB) [file 13428_2024_2345_MOESM1_ESM.docx]

**S1. Systematic Review Search strategies used per database**

*PubMed*

(("intru*"[Title/Abstract] OR "unwanted"[Title/Abstract] OR "involuntary"[Title/Abstract]) AND ("memory"[MeSH Terms] OR "memor*"[Title/Abstract]) AND ("suppress*"[Title/Abstract] OR "control"[Title/Abstract] OR "regulat*"[Title/Abstract])) AND (2012:2023[pdat])

*Web of Science*

(TS=("intru*" OR "unwanted" OR "involuntary") AND TS=("memor*") AND TS=("suppress*" OR "control" OR "regulat*")) and 2023 or 2022 or 2021 or 2020 or 2019 or 2018 or 2017 or 2016 or 2015 or 2014 or 2013 or 2012 (Publication Years) and Computer Science Information Systems or Engineering Electrical Electronic or Computer Science Theory Methods or Telecommunications or Computer Science Software Engineering or Computer Science Artificial Intelligence or Computer Science Hardware Architecture (Exclude – Web of Science Categories)

*PsychInfo*

(TI ( "intru*" OR "unwanted" OR "involuntary" ) OR AB ( "intru*" OR "unwanted" OR "involuntary" ) OR KW ( "intru*" OR "unwanted" OR "involuntary" )) AND (( DE "Memory" OR DE "Associative Memory" OR DE "Autobiographical Memory" OR DE "Collective Memory" OR DE "Early Memories" OR DE "Eidetic Imagery" OR DE "Episodic Memory" OR DE "Explicit Memory" OR DE "False Memory" OR DE "Forgetting" OR DE "Implicit Memory" OR DE "Long Term Memory" OR DE "Memory Consolidation" OR DE "Memory Decay" OR DE "Memory Trace" OR DE "Prospective Memory" OR DE "Reminiscence" OR DE "Repressed Memory" OR DE "Retrospective Memory" OR DE "Short Term Memory" OR DE "Spatial Memory" OR DE "Spontaneous Recovery (Learning)" OR DE "Tip of the Tongue Phenomenon" OR DE "Verbal Memory" OR DE "Visual Memory" ) OR TI memor* OR KW memor* OR memor*) AND (TI ( suppress*" OR "control" OR "regulat*" ) OR AB ( suppress*" OR "control" OR "regulat*" ) OR KW ( suppress*" OR "control" OR "regulat*" ) )

**S2: PRISMA Flowchart (Page et al., 2021) of the literature search and exclusion of studies.**

**Identification of studies via databases**

Records removed *before screening*:

Duplicate records removed

(n = 669)*

Records identified from*:

Databases (n = 1997)

**Identification**

Records screened

(n = 1328)

Records excluded

(n = 1210)

Reports sought for retrieval

(n = 118)

Reports not retrieved

(n = 0)

**Screening**

Reports assessed for eligibility

(n = 118)

Reports excluded:

Did not use T/NT task (n = 23)

Did not have an intrusion measure (n = 73)

Studies included in review

(n = 22)

**Included**

*A semi-automatic tool was used to detect duplicates. Any matches > 90% were automatically removed. Articles with matches < 90% (n = 37) were checked manually.

**The association between intrusion control and suppression-induced forgetting**

To compare the slope approach to the IIC and explore their relation to SIF, we performed correlation analyses for each available dataset (see Table S1). To give an overview of different approaches employed by previous studies (see Table 1 in the main text), we calculated 4 slope measures in total: 1) a beta coefficient across all blocks of the T/NT (Slope_all_): 2) a proportionalised version of Slope_all_, calculated by dividing by the frequency of the first block (Slope_all_); 3) a beta coefficient across condensed blocks (Slope_condensed_); 4) a proportionalised version of Slope_condensed_, calculated by dividing by the frequency of the first block (i.e. the average frequency of blocks 1 and 2). Previous studies (Ashton et al., 2020; Hellerstedt et al., 2016; Quaedflieg et al., 2022) have calculated a SIF_base_ index by subtracting the recall of No-Think items from Baseline items and then dividing by Baseline items ((Baseline – No-Think) / Baseline). This results in a subject-specific measure of forgetting relative to baseline memory performance, with higher positive index scores indicating increased forgetting. We included this measure as well as SIF calculated as Baseline – No-Think, with higher positive scores indicating increased forgetting.

**Table S1: Correlations between IIC, linear slope (all and condensed blocks) and SIF indexes with and without baseline correction.**

|  |  | |  |  | SIF | | | | | | | | | | SIF_Base_ | | | | | | | | | |
| --- | --- | --- | --- | --- | --- | --- | --- | --- | --- | --- | --- | --- | --- | --- | --- | --- | --- | --- | --- | --- | --- | --- | --- | --- |
|  |  | |  |  |  | | Slope with baseline correlation (i.e. proportionalised) | | | | Slope without baseline correction | | | |  | | Slope with baseline correlation (i.e. proportionalised) | | | | Slope without baseline correction | | | |
|  |  | |  |  | IIC | | Slope_all_ | | Slope_condensed_ | | Slope_all_ | | Slope_condensed_ | | IIC | | Slope_all_ | | Slope_condensed_ | | Slope_all_ | | Slope_condensed_ | |
| Paper | Valence | | *N* | SIF | *r* | *p* | *r* | *p* | *r* | *p* | *r* | *p* | *r* | *p* | *r* | *p* | *r* | *p* | *r* | *p* | *r* | *p* | *r* | *p* |
| Ashton et al. (2020) | Autobiographic future fears | | 40 | Y | .073 | .65 | .23 | .16 | .20 | .23 | .25 | .11 | .26 | .10 | .070 | .67 | .32 | .052 | .23 | .15 | .38 | .017* | .38 | .015* |
| Ashton et al. (2023) | Neutral | | 75 | Y | -.03 | .80 | 0.03 | .80 | .04 | .73 | .018 | .88 | .012 | .92 | -.025 | .83 | .032 | .79 | .046 | .70 | .017 | .89 | .010 | .93 |
| Chen et al. (2022) | Neutral | | 42 | Y | -.25 | .11 | -.52 | <.001* | -.46 | .002* | -.44 | .003* | -.44 | .003* | -.21 | .19 | -.48 | .001* | -.43 | .005* | -.39 | .011 | -.39 | .012* |
| Davidson et al. (2020) | Neutral | | 17 | Y | -.42 | .11 | -.42 | .12 | -.13 | .63 | -.22 | .39 | -.23 | .38 | -.39 | .13 | -.38 | .16 | -.11 | .67 | -.20 | .43 | -.21 | .42 |
|  | Negative | | 17 | Y | -.40 | .13 | -.28 | .32 | -.27 | .29 | -.39 | .12 | -.36 | .15 | -.33 | .21 | -.23 | .40 | -.24 | .35 | -.33 | .20 | -.30 | .25 |
| Legrand et al. (2020): Study 1 | Neutral | | 28 | Y | -.31 | .11 | -.30 | .12 | -.16 | .41 | -.36 | .62 | -.38 | .050* | -.27 | .16 | -.30 | .188 | -.14 | .47 | -.33 | .091 | -.33 | .085 |
|  | Disgust | | 28 | Y | .086 | .66 | -.21 | .35 | -.22 | .29 | .024 | .91 | .002 | .99 | -.013 | .95 | -.18 | .40 | -.28 | .16 | -.056 | .77 | -.072 | .72 |
|  | Sad | | 28 | N | .29 | .14 | -.16 | .43 | -.057 | .78 | .087 | .66 | .038 | .85 | .32 | .097 | -.10 | .61 | .007 | .97 | .08 | .69 | .05 | .79 |
| Legrand et al. (2020): Study 2 | Neutral | | 24 | N | -.21 | .33 | .088 | .69 | .087 | .69 | -.20 | .34 | -.25 | .24 | -.22 | .30 | .082 | .71 | .085 | .70 | -.21 | .33 | -.25 | .24 |
|  | Emotional | | 24 | N | -.10 | .64 | .13 | .55 | .066 | .76 | -.025 | .91 | -.032 | .88 | -.11 | .62 | .122 | .57 | .059 | .78 | -.032 | .88 | -.040 | .85 |
| Liu, et al. (2020) | Neutral and Negative combined | | 27 | N | -.30 | .13 | -.49 | .010  * | -.50 | .008* | -.53 | .004* | -.55 | .003* | -.32 | .10 | -.49 | .009* | -.50 | .008* | -.53 | .004* | -.56 | .003* |
| van Schie & Anderson (2017) | Same probe | Short suppression trial | 40 | Y | .20 | .22 | .34 | .033  * | .40 | .012 | .30 | .056 | .26 | .11 | .21 | .20 | .41 | .009* | .35 | .028* | .31 | .054 | .26 | .10 |
|  |  | Long suppression trial | 40 | Y | .15 | .34 | .081 | .62 | .16 | .34 | .11 | .49 | .12 | .45 | .16 | .33 | .075 | .65 | .15 | .35 | .11 | .52 | .11 | .49 |
|  | Independent probe | Short suppression trial | 40 | Y | .033 | .84 | .16 | .31 | .16 | .32 | .16 | .32 | .14 | .39 | .048 | .77 | .13 | .44 | .13 | .43 | .14 | .38 | .12 | .47 |
|  |  | Long suppression trial | 40 | Y | .37 | .019* | .27 | .09 | .29 | .068 | .36 | .021* | .37 | .017* | .33 | .038* | .25 | .12 | .26 | .099 | .33 | .035* | .34 | .032* |
|  |  |  |  |  |  |  |  |  |  |  |  |  |  |  |  |  |  |  |  |  |  |  |  |  |

Whether the study found a significant SIF effect (Yes/No) is outlined per condition. The SIF is calculated as the difference in recall between Baseline – No-Think. SIF_Base_ is calculated as (Baseline – No-Think)/Baseline. Negative correlations indicate that increased intrusion control (slope or IIC) is associated with an increased forgetting effect. Positive correlations indicate that increased intrusion control is associated with a decreased forgetting effect. *Indicate significance level p < .05.

All studies presented calculated SIF from cued-recall or recognition tasks. For Nishiyama and Saito (2022), no SIF values are included as only reaction times were available for the recognition task. For Mary et al. (2020), no SIF values are included as the study used a priming task. For Satish et al., (2022), no SIF values are included as a baseline condition was not used.

**References**

Ouzzani, M., Hammady, H., Fedorowicz, Z. & Elmagarmid, A. (2016). [Rayyan — a web and mobile app for systematic reviews](http://rdcu.be/nzDM). *Systematic Reviews, 5*, 210. DOI: 10.1186/s13643-016-0384-4.

Page, M.J., McKenzie, J.E., Bossuyt, P.M., Boutron, I, Hoffmann, T.C., Mulrow, C.D., et al. (2021). The PRISMA 2020 statement: an updated guideline for reporting systematic reviews. *BMJ, 372*: n71. doi: 10.1136/bmj
